# Reports of Cases Occurriug in the Medical Ward of the Buffalo Hospital

**Published:** 1857-11

**Authors:** A. W. Nichols

**Affiliations:** Assistant to Chair of Clinical Medicine, University of Buffalo


ART. IV.—*Reports of Cases treated in the Male Medical Ward of the Buffalo Hospital of the Sisters of Charity*, AUSTIN FLINT, M. D., *Attending Physician, during the College Session of* 1856–7, *being from October* 1, 1856 *to February* 18, 1857. Reported by A. W. NICHOLS, M. D., Assistant to Chair of Clinical Medicine, University of Buffalo.

No. VIII. *Acute Lobar Pneumonitis. Treatment,—Opiates and Stimulants.*

It is not intended, in this article, to enter into a discussion of the various methods of treatment employed in acute lobar pneumonitis. That there is a remarkable diversity of opinion in regard to the treatment of this disease in our latitude, is well known. And as we are about to bring forward the methods of treatment employed and the results afforded in a few cases of this disease, it should be remembered, that it is not our intention to proscribe other plans of treatment such as frequently have been advised and adopted. Whatever may be the conclusions we form, of the disease under consideration, it should ever be borne in mind that pneumonitis may occur as an idiopathic or symptomatic affection. Does it not often exist as a symptomatic disease, due perchance to a morbid condition of the blood, and much more frequently than is generally supposed?

Besides, it should be remembered, that mere recollections of the results of any peculiar method of treatment, are more or less influenced by the opinions entertained of the nature of the disease and the success attending the employment of the favorite remedies, in a greater or less number of instancs, as the case may be.

Those conclusions, however, which are derived from cases duly recorded, are such as no previous bias of the mind can affect: and by means of them, the preconceived value of favorite plans of treatment is either confirmed or disproved. We desire, at present, to direct the attention of the reader, to the course, termination, and treatment pursued in eleven cases of this disease which were admitted into the hospital, during the past winter and spring.

All the patients were adult males; the youngest being 19 and the eldest 36 years of age. In two cases, the disease occurred in connection with prior tuberculous deposit, probably. In one case, the patient had been suffering from an attack of intermittent fever. And in the remaining eight cases, the disease was uncomplicated or simple.

In these eleven patients, with a single exception, the disease terminated in recovery; in this instance, the pneumonitis was progressing favorably, when the sudden supervention of other disease, probably pericarditis, caused the death of the patient.

Of the eight uncomplicated cases, all of which ended in speedy recovery, in one the inflammation was latent and no diagnosis was made until the eighth day after the attack; the entire right lung being affected, and yet no symptom was presented of sufficient importance to direct attention to the chest; this patient was up and walking about the ward most of the day. In another case admitted eight days after date of attack, symptoms existed closely resembling those presented in typhoid fever; this patient was treated with those remedies ordinarily made use of in the latter disease, and rapidly improved under such treatment.

Fonr other cases presented delirium manifested by incoherent talking and muttering, and by attempts to get out of bed: the delirium in one of these cases may have been due to an absence of accustomed stimulus. In one case, there was no delirium present nor any unusual phenomenon of this disease. And in the remaining case, the inflammation had ceased before admission to the ward.

In four of the foregoing eight cases, the patients had no medical treatment prior to their admission. One of these four, entered on the sixth day, two on the eighth day, and one on the twelfth day after the date of attack; the latter patient was convalescent when admitted. In one other patient, the only treatment which had been made use of, was the application of large sinapisms to the affected side, which produced extensive vesication; no less than seventy square inches of the cutaneous surface having been blistered at the same time.

Of the two cases of pneumonitis supervening upon tuberculosis probably, both had had previous medical treatment: after their admission, they were placed on the use of cod liver oil, sulphate of quinia, and a sustaining diet, consisting of essence of beef and milk porridge, with a small quantity of brandy. One of these patients was pronounced convalescent on the eighth day, and returned to work on the seventeenth day after the date of attack. In the other patient who had been bled before his admission, convalescence took place on the fourteenth day, and he left the hospital on the twenty-second day after the date of attack.

Of the remaining nine cases, four had medical treatment prior to their admission: one of these patients who had had intermittent fever, was bled and blistered before he entered the hospital. The treatment of these nine patients after entering the ward, was as follows: brandy and nutritious diet, with or without Dover's powder, in two cases; sulphate of quinia, with cod liver oil or with Dover's powder, in two cases; brandy, accompanied with opium in large doses, in one case; tartrate of antimony and potassa, followed by opium in large doses, in one case; opium in large doses, in two cases; and Dover's powder in the case convalescent on admission.

In the two cases in which a diffusible stimulant, such as brandy, was the main article of treatment: no medical treatment had been received prior to their admission. Delirium existed in both cases. One of these patients, who presented symptoms resembling those of typhoid fever, was treated with brandy f. ℥ss, every three hours, and Dover's powder; immediate improvement was observed, and convalescence took place on the fourteenth day after date of attack; and twenty days thereafter, he was discharged from treatment. The other patient entered on the sixth day after the date of attack, with delirium, the pulse being 72, and the respirations 20; physical signs showed inflammation of the lower lobe of left lung. This patient was placed on the use of brandy f. ℥ss, every three hours, with the sustaining diet previously mentioned. He progressively and rapidly improved; on the eleventh day after the date of attack, he was convalescent; and on the next day, he walked away from the hospital.

Of the four cases in which opium was chiefly relied on, three had received medical treatment before their admission. Delirium existed in three of these four patients. In that case in which brandy was employed, accompanied with large doses of opium, the patient entered five days after date of attack; the lower lobe of the left lung was in the second stage of the disease or solidified. Alternately with a small quantity of brandy, two grains of opium every three hours, were given. The patient improved daily; and on the eleventh day after date of attack, he was convalescent. In that case in which antimony was first given and followed by opium in large doses, the patient had been extensively vesicated on the affected side, by the application of mustard. He entered on the seventh day after date of attack; the lower lobe of the left lung was solidified. After the use of antimony for two days, opium in doses of one grain every three hours, was given. Convalescence took place on the sixteenth day after date of attack.

In order to show more fully the beneficial effects attending the use of brandy in the two cases in which it formed the chief means of treatment, we will introduce these cases, merely presenting the symptoms afforded on the day the brandy was employed and on the day or days succeeding its administration.

Likewise, in the two cases in which opium was the main article of treatment, we will present the symptoms on the day the opium was administered and on the day following, illustrating, thereby, in a striking manner, the favorable change in the condition of these patients, resulting from the use of this medicine.

A few words, however, are necessary in reference to the expressions "date of attack" and "period of convalescence" employod in the preceding analysis. By the term "date of attack," is intended to express the very first manifestations of disease, such as the chill followed by febrile movement, or vomiting, or lancinating pain commonly referred to the region of the nipple, or cough. These phenomena may be presented some days previous to any *marked* symptoms referrible to the chest, or before the patient is obliged to take to the bed. By the term "period of convalescence," we mean, that particular time at which the patient exhibited *marked* improvement. This may be before the patient is able to be out of bed, as generally is the case; but, it may not be observed until a few days after the patient has been up and dressed. In pneumonitis, there is frequently an indisposition to take to the bed, and a great desire to get up, even during the height of the inflammation. In one of the foregoing cases, the patient continued to walk about the ward most of the day, although the entire right lung was inflamed. And in another instance, the patient persisted in the attempt to rise from the bed and dress himself, although the lower lobe of one lung was then solidified.

*Two Cases of Simple Acute Lobar Pneumonitis, showing the beneficial effects of Brandy.*

Case A. March 18th, W. N., aged 19, admitted on the eighth day after date of attack, having had no previous medical treatment. Aspect dull; considerable capillary congestion of face but diffused, also of backs of hands. Answer questions readily and intelligibly. Complains of thirst, and of pain in lower portion of left side of chest, and in the right iliac region. Has slight cough and moderate expectoration of opaque, rusty sputa. Respirations 40; pulse full, but compressible, numbering 108. Skin hot and dry; tongue protruded slowly and withdrawn slowly, moist and coated; sordes on the teeth and lips. Has no headache, no tinnitus aurium. Has no nausea, nor vomiting, nor diarrhœa. Has no appetite. Tenderness on pressure in right iliac region; no eruption present. Lies on the back, and sleeps most of the time. Slight manifestations of delirium. (Case supposed to be one of typhoid fever.)

*Treatment.* Brandy and water, of each f. ℥ss, every three hours; cough syrup f. ʒj; and Dover's powder, grs. x; cold to the head, and sustaining diet.

March 19th. Brandy had been faithfully administered. Rested well during the night; no delirium; less capillary congestion of face. Answers questions readily and intelligibly. Complains of no pain in chest, except during the act of coughing, and then in the same situation as yesterday. To-day, has not much cough and very little expectoration of sputa, same in character as yesterday. Skin cool and moist. Respiration 24; and pulse 80. Tongue coated, moist, but protruded readily; sordes on teeth and lips. Has no appetite. Tenderness in right iliac region still continues; has no diarrhœa; no eruption present.

(Above record, diagnosis, and treatment, by Nichols.)

This morning, (the chest being examined by Dr. Flint,) unmistakable evidence of inflammation of the lower lobe of left lung were afforded.

The same treatment was continued; and speedy convalescence took place.

In this case, under the use of brandy, observe the daily improvement of the patient; the typhoid symptoms disappearing rapidly. Notice also the reduction of the pulse: first day, 108; second day, (brandy having been given,) 80; next record, 76; day of convalescence, 64. Likewise, the respirations were diminished: first day, 40; second day, 24; next record, 20.

Case B. November 25th, I. B., aged 23, admitted on the sixth day after date of attack, having had no prior medical treatment. Record, two days after admission. Has been delirious at night and during part of yesterday, talking incoherently. Was placed yesterday on the use of brandy f. ℥ss, every three hours, and sustaining diet. This morning, he reports much better. His mind is perfectly clear. Pulse 72 and respirations 20. Skin warm and mellow. Frequent cough with bloody expectoration small in amount.

An examination of the chest disclosed inflammation of lower lobe of left lung in its second stage.

The same treatment, and only that, was continued. Progressive and rapid improvement took placé; and on the fifth day after his admission, he was convalescent.

(Above record by Nichols.)

In this case the sudden disappearance of the delirium, under the use of brandy, is worthy of observation. The improvement in other respects, was as rapid as in the first instance, if not more so.

*Two Cases of Simple Acute Lobar Pneumonitis illustrating the marked improvement of the Patients under the use of large doses of Opium.*

Case A. June 8th, M. D., aged 36, has been addicted to intemperance of late; presents a pallid and morbid aspect; is considerably emaciated. Was attacked with chills, &c., on 28th ult; pain, cough, and expectoration, first occurred on the 1st inst.

"He now presents an appearance of anxiety and prostration. The respirations are 42. The alæ nasi dilate moderately. Pulse 120, quick and vibratory. Tongue thinly coated, dry and swelled at the extremity. Sordes on the teeth. No delirium. Expectoration, tenacious, not opaque, not abundant, presenting a reddish yellow color, pretty uniformly diffused."

The physical signs, on examination of the chest, showed inflammation of the entire right lung.

The treatment was gum opium, two grains every six hours, and a small quantity of ale.

The next day, the record runs thus: "His aspect is greatly improved. Pulse 104, more volume and force; respirations 26."

June 11th. Reports better and aspect greatly improved. Pulse 80; respirations 28 and not labored. The tongue is clear and somewhat dry. No delirium. No pain, except in both shoulders and arms. Expectoration adhesive, not opaque, and greenish. Dejection this morning. Has some appetite."

The same treatment had been continued.

This patient rapidly improved, and was convalescent ten days after he entered the hospital.

Case B. July 2d, I. M., aged 32, had intermittent fever a year ago; but since his present illness, has not had any chills, with rigors, till yesterday.

He was admitted this morning, and appeared to be in the cold stage of an intermittent, having chills and severe rigors.

"He has been confined to his bed about twelve days. He was attacked suddenly with pain in the side, and fever. His mind is apparently dull and confused. * * * * He has been bled, and very extensively blistered over the chest in front and on the right side. The pulse is now 145, and rather full. The skin is warm. Tongue thickly coated. Respirations 36. He has cough and expectoration, character of the latter not observed. He states, that he is a very hard drinker, and has of late drank very freely."

A physical examination of the chest disclosed inflammation affecting the entire right lung.

The treatment was: sulphate of quinia, grs. v, and pulv. opii, grs. ij, every four hours; brandy f. ℥j, every four hours, with sustaining diet.

The following day, "He reports much better; and his aspect was greatly improved. Passed a quiet night; no manifestations of delirium. Pulse 120, with considerable volume; respirations 32. Has had several small dejections. Tongue coated in centre and clean at edges. Has no appetite, but complains of thirst. The cough is not frequent or severe, and expectoration small. A single sputum examined, is distinct and opaque, without any rusty tinge."

The next day, improvement continues. "Pulse 100, and respirations 26. Has had several small evacuations since the last record."

In these two cases, the rapid improvement of the patients under the use of large doses of opium, *from eight to twelve grains in twenty-four hours*, was very striking.

The effect on the pulse and respirations, is to be noticed. In the first case, on day of admission, the pulse was 120; respirations 42. He was placed on the use of opium. On the second day the pulse fell to 104, and the respirations to 26. Third day, pulse 80; respirations 28, but not labored.

In the second case, on the day of admission, the pulse numbered 145, and the respirations 36. The use of opium having been commenced, on the second day, pulse 120; respirations 32. Third day, pulse 100; respirations 26.

In this case it will be observed, that *diarrhœa* continued, although the patient was taking two grains of opium every four hours.

*Addenda.* The following case of acute lobar pneumonitis affecting the lower lobe on the left side and the middle lobe on the right side, has come under observation since writing the foregoing. This patient is now under treatment at the hospital, (Oct. 13th.) As this case illustrates in such a striking manner, the marked improvement following the use of morphia and a small quantity of stimulus, that we leave to give the case in in detail:

F. B., aged 26 years, was admitted to hospital on 26th of Sept., 1857. Dr. Flint's service commenced on the 1st of October.

The first record of this case was not made until the 5th of October, owing to the number of patients in the ward, the commencement of new services, &c. On that day, the patient presented over the right side of chest and abdomen, and over the lumbar region, a pustular eruption, not very copious, discrete, and presenting all the characteristics of ecthyma. He had had since the 1st of Oct., a cough, and was unable to speak above a whisper; but his cough was less urgent and his speech was returning.

On the 6th, the record runs thus: "He has now recovered his voice and is not hoarse. Cough and expectoration continue. He had yesterday a paroxysm of fever; a chill, febrile movement, and sweating. To-day he has diarrhœa."

There appeared nothing worthy of record in this case until the 10th of Oct. The patient keeping the bed all the time, and reporting about the same each day.

Oct. 10th. Yesterday, about 12 o'clock, this patient was suddenly attacked with pain in the region of the right nipple; the pain was sharp and lancinating, and obliged him to suppress his breathing. He had a chill, vomited, and lost his appetite; cough and expectoration appeared.

This morning, he appears to be delirious; was talking incoherently durthe night. (Attendant states that he has been delirious for the past two nights.) There is slight circumscribed capillary congestion of cheeks; face otherwise pallid. Complains of pain, sharp and severe, in both sides of chest, but most in the right; the pain is increased by the acts of coughing and forced breathing. Cough frequent and painful; expectoration copious, and chiefly of clear mucus, not rusty. *Respirations* 36; *pulse* 102. Skin warm and mellow.

The weakness of the patient prevented a prolonged examination of the chest. The following is the record by Prof. Flint: "This patient presents notable dullness on percussion, and bronchial respiration over the lower lobe of left lung. He also presents on the right side, over lower scapular region, bronchial respiration; but not below. On both sides, dry and moist mucous râles. The crepitant râle was not observed; but owing to feebleness and irritability, he was not examined minutely. He was probably attacked with pneumonitis affecting the left lung, on the 6th inst. The symptoms did not direct attention to the chest, and a physical examination was not made. He has now pneumonitis in the second stage, affecting the lower lobe of left lung; and pneumonitis on the right side, commencing yesterday, which has not yet involved the whole of the lower lobe.* Bronchitis coëxists."

The treatment was morphiæ sulph., gr. ¼, every six hours.

Oct. 11th. On the evening of the 10th, this patient was visited by myself. He was actively delirious, talking incoherently, raising himself up, and attempting to get out of bed. *His respirations were* 36, *and pulse* 100. Skin bathed in perspiration. As the patient appeared to be rapidly sinking, and the delirium being active, the treatment of the morning was slightly modified. The *morphia was increased* to gr. ½, every four hours; and *whisky* f. ℥ss, every three hours.

This morning there is a *marked improvement.* The aspect is much better. *The mind is clear. The respirations are* 24, *and the pulse* 80. The expectoration was quite abundant, and highly characteristic: it cannot well be described. Physical signs about the same; the patient's condition preventing an extended examination of the chest.

*Treatment.* Morphiæ sulph., gr. ¼, every six hours; and whisky f. ℥ss, every four hours; with sustaining diet.

Oct. 12th. Reports much better. Slept most of the night. Was delirious for a short time, however. This morning, his mind is clear; aspect much improved; answers questions correctly; is not as irritable as he was yesterday morning; is disposed to sleep, when not disturbed. No capillary congestion of cheeks; face pallid; tongue moist and coated. Skin cool, and bathed in perspiration. *Respirations* 20; *pulse* 76. Cough frequent; expectoration more clear, less rusty.

*Treatment.* Morph., gr. ¼, every eight hours; and *omit whisky.*

The prognosis in this case, is favorable; the result will be given hereafter.

Excluding this case, and the two cases in the foregoing report, which were probably complicated with prior tuberculous deposit, both of which left the hospital cured of the pneumonitis, there occurred but one death in the remaining nine cases. It is a noteworthy fact, that these twelve cases include all the patients treated for pneumonitis during Prof. Flint's service, for a

* In a day or two after this record, it was discovered that the inflammation of the right lung was confined to the middle lobe.

period of—not more than—thirteen months altogether.† The total number of patients treated during the last college session, a period of about twenty weeks, from the beginning of October to the middle of February, was 128: of this number, seven were cases of pneumonitis, five of these being cases of simple acute lobar pneumonitis. The proportion of cases of this disease to the whole number of cases treated, was quite small, less than four per cent.

In not one of the twelve cases, did blood-letting, general or local, form a part of the treatment after their admission to the hospital; and in only two or three instances, did it enter into the treatment prior to their admission. This is offered simply as a fact; no influence prejudicial to the employment of depletion generally or locally, should be drawh from the above statement; neither must it be considered as an expression of Prof. Flint's views respecting the utility of depletion in this disease.

It will be noticed that some of these cases received no active treatment' being brought to the hospital or found in the ward with physical signs retrospective of acute lobar pneumonitis. A few cases were treated by stimulants; whilst others proceeded to a favorable termination under the use of opiates. No case illustrating the beneficial effects of opium or its preparations, in a more striking manner than the last instance.

† That is, from April, 1856 to May, 1857, and part of October, 1857.

